# Sir Ernest Cassel's Further Gifts to Hospitals

**Published:** 1915-12-25

**Authors:** 


### SIR ERNEST CASSEL'S FURTHER GIFTS TO HOSPITALS.

The £50,000 4½ per Cent. War Loan Stock which Sir Ernest Cassel is giving to hospitals and kindred charities is to be distributed on the same general lines as his previous donation of £50,000 in 1911—that is, partly in direct gifts and partly through King Edward's Hospital Fund. £22,000 has accordingly been allocated by Sir Ernest Cassel as follows, viz.:—

Gifts of 4½ per Cent. War Loan Stock (Nominal Value) made by the Right Hon. Sir Ernest Cassel, G.C.B., G.C.M.G., G.C.V.O.

### 1. To London Hospitals as follows:

| | £ |
|---|---|
| London | 800 |
| King's College | 500 |
| Guy's | 1,000 |
| St. Mary's | 300 |
| Royal National Orthopædic | 600 |
| West London | 600 |
| *Dreadnought* | 700 |
| Hampstead General | 100 |
| London Lock | 100 |
| Charing Cross | 750 |
| City of London Hospital for Diseases of the Chest | 200 |
| Great Northern Central | 400 |
| Hospital for Consumption, Brompton | 250 |
| Middlesex | 500 |
| Prince of Wales's | 600 |
| University College | 600 |
| Hospital for Sick Children, Great Ormond Street | 300 |
| Metropolitan | 300 |
| Queen's Hospital for Children | 300 |
| Westminster | 400 |
| Royal Free | 400 |
| Miller General Hospital for South-East London | 400 |
| Mount Vernon Hospital for Consumption | 200 |
| Royal London Ophthalmic | 50 |
| East London Hospital for Children | 200 |
| West Ham and Eastern General Hospital | 100 |
| St. Mary's Hospital for Women and Children, Plaistow | 50 |
| Hospital for Women, Soho Square | 550 |
| St. Bartholomew's | 1,000 |
| Queen Charlotte's Lying-in | 250 |
| National Hospital for the Paralysed and Epileptic | 250 |
| St. Thomas's | 750 |

### 2. To Country Hospitals as follows:

| | £ |
|---|---|
| Addenbrooke's Hospital, Cambridge | 1,000 |
| Victoria Hospital, Bournemouth | 1,000 |
| West Suffolk Hospital, Bury St. Edmunds | 1,000 |
| Romsey Nursing Home, Romsey, Hants | 500 |
| Royal Hants County Hospital, Winchester | 1,000 |
| Victoria Hospital, Blackpool, Lancs | 1,000 |
| Fleetwood Cottage Hospital, Fleetwood, Lancs | 500 |
| Lytham Cottage Hospital, Lytham, Lancs | 500 |
| Royal National Hospital for Consumption, Ventnor | 1,000 |
| Royal Sea-Bathing Hospital, Margate | 1,000 |
| Total | £22,000 |

### 3. £28,000 through the King's Fund.

As was announced at the meeting of the General Council of King Edward's Hospital Fund on December 16, the remaining £28,000 has been entrusted by Sir Ernest Cassel to the Fund for the purpose of increasing by one-fifth the grants already made by the Fund this year.

